# Isolation and identification of a novel canine parvovirus type 2c strain in domestic cats in Dalian, China

**DOI:** 10.3389/fvets.2022.1001604

**Published:** 2022-10-13

**Authors:** Zheng Jing, Peng Ji, Yanquan Wei, Fuxing Hao, Yanming Wei

**Affiliations:** ^1^College of Veterinary Medicine, Gansu Agricultural University, Lanzhou, China; ^2^Jiangsu Agri-Animal Husbandry Vocational College, Taizhou, China

**Keywords:** canine parvovirus, feline panleukopenia virus, domestic cats, *VP2* gene, CPV-2c strain

## Abstract

Canine parvovirus (CPV) and feline panleukopenia virus (FPV) are highly contagious and cause severe enteric diseases, with high mortality rates in dogs and cats. In the present study, we isolated and identified a novel CPV-2c strain (FPV-DL04 strain) from 18 cats with gastroenteritis symptoms and a positive parvovirus PCR test result in Dalian, China. Molecular characterization, sequence analysis, and phylogeny determination were performed on the *VP2* gene of this strain. The results showed that the FPV-DL04 strain had 99.4% homology with the CPV-2c CN/HN1708 strain, and both strains had S297A and A300G key mutation sites. Interestingly, we also found that the DL04 strain has a A5G mutation site, but no F267Y and Y324I mutation sites. This study provided new important findings regarding the evolution of parvovirus infection in domestic cats in China.

## Introduction

The family *Parvoviridae* comprises small, non-enveloped, single-stranded DNA viruses with a 4–6 kb linear genome that infect a wide range of animals. Canine parvovirus (CPV or CPV-2) and feline panleukopenia virus (FPV) are variants of Carnivore protoparvovirus 1, which belong to the genus *Protoparvovirus* in the family *Parvoviridae* (1, 2). These viruses cause a variety of severe diseases, especially in young animals (3). The parvovirus genome consists of two open reading frames (ORFs) that encode for structural proteins (VP1 and VP2) and non-structural proteins (NS1 and NS2) (1).

Feline panleukopenia virus and CPV are highly contagious and can cause high mortality and severe hemorrhagic gastroenteritis in cats and dogs (4). Canine parvovirus type 2 (CPV-2) variants was first reported in 1977 (5), and several studies have found that CPV-2 variants originated from FPV with at least six coding nucleotide differences in the *VP2* gene (6). While dogs are susceptible only to the original CPV-2 and all its variants, cats are susceptible to both FPV and CPV-2 variants, except the original CPV-2 type (7). According to the variation in the VP2 antigen, CPV-2 variants is classified into three antigenic types (CPV-2a, CPV-2b, and CPV-2c). CPV-2a and CPV-2b have 426N and 426D unique amino acids, respectively (7). In 2000, CPV-2c was first discovered in Italy with the unique amino acid 426E (8). At present, CPV-2c is circulating in Europe, America, and Asia (8–10). In 2010, CPV-2c was first reported in China, and many of the CPV-2c strains reported subsequently were mainly derived from dogs (11). Until 2022, a CPV-2c strain derived from domestic cats was first reported in Beijing, China (12).

In recent years, several studies have reported the spread of CPV-2 in dogs in some provinces of China. However, there is limited information on the transmission of FPV and CPV-2 in domestic cats; hence, epidemiological investigations are needed to assess the transmission and evolution of parvovirus in domestic cats in China. In the present study, we investigated the prevalence of parvovirus in domestic cats in Dalian, China. We characterized the nucleotide sequences and key amino acid sites of the *VP2* gene of FPV and CPV-2 collected from domestic cats. We isolated and identified a CPV-2c genotype strain (FPV-DL 04) and found a new mutation site (A5G) in this strain. This study will provide important clues for investigating the prevalence of parvovirus in northeast China and to gain insights into the evolution of CPV and FPV.

## Materials and methods

### Clinical sample collection

Between 2017 and 2021, 50 clinical samples of rectal swabs were collected from domestic cats with feline diarrheal disease characterized by depression, anorexia, vomiting and diarrhea at a pet hospital in Dalian, northeast China. The rectal swab samples were sent to the laboratory for diagnosis. The fecal samples were homogenized in 1 ml of 0.1 M PBS (pH 7.4), centrifuged at 10,000 *g* for 10 min at 4°C, and stored at −80°C until analysis.

### Parvovirus screening and identification

DNA was extracted from each sample using the DNeasy Blood and Tissue Kit (Qiagen, Germany) in accordance with the manufacturer's instructions. The presence of FPV/CPV in the extracted DNA was screened by PCR using the primers pv-F (5′-TACAGGATCTGGGAACGGGT-3′) and pv-R (5′-GCCATGTATGTGTTAGTCTACATGG-3′) amplifying a 779-bp fragment of a part of the *VP2* gene. The primers were designed for the conserved sequences of FPV and CPV. In brief, PCR was performed in a 20-μl reaction mixture consisting of 10 μl 2 × Taq Plus PCR Master Mix (Vazyme, China), 0.5 μl of each primer, 7 μl of nuclease-free water, and 2 μl of DNA template. Distilled water served as a negative control. The PCR conditions were set as follows: denaturation at 94°C for 3 min; 32 cycles of denaturation at 94°C for 30 s, annealing at 54°C for 45 s, and extension at 72°C for 30 s; and final extension at 72°C for 5 min. The expected size of the parvovirus-positive amplified product was 799 bp. The amplified products were electrophoresed on a 1% agarose gel, and the bands were observed in a Tanon 1600 imaging system (Tanon, China).

### VP2 gene sequencing

For samples positive for parvovirus, the full-length *VP2* gene was amplified using the primers VP2-F (5′-ATCTTGCACCAATGAGTGATG-3′) and VP2-R (5′-CTAGGTGCTAGTTGATATGTAATAAAC-3′). The primers were designed for the conserved sequences of parvovirus. The expected size of the amplified product was 1,813 bp. The purified PCR product was ligated into the pMD18-T vector (Takara, Japan) and transformed in *Escherichia coli* DH5α competent cells (TransGen Biotech, China). The positive plasmid was identified and then sent to Beijing Qingke Biotechnology Co., Ltd. for sequencing. The sequencing results were spliced and analyzed using the DNA Star software package.

### Sequence analysis

The obtained VP2 amino acid sequence and the reference sequences were used for homology analysis, alignment, and typing using MEGA 7.0 software. Test sequences were typed by analyzing specific mutation sites on VP2 in FPV/CPV and CPV variants. Subsequently, the protein structure of VP2 was predicted online using the Swiss Model software (https://swissmodel.expasy.org) (13), and the CPV VP2 structure (PDB ID: 4dpv.1) (14) was used as the template. The predicted VP2 protein structure was analyzed using the Swiss-PDB Viewer software, and the mutation sites were displayed.

### Phylogenetic analysis

To elucidate the evolutionary history of FPV VP2 sequences identified in this study, a phylogenetic tree was constructed with 18 sequences obtained in this study and 41 sequences corresponding to the full-length *VP2* gene from CPV and FPV strains (GenBank Accession No. FPV: EU498695.1, EU498711.1, EF418569, EU498712.1, EU498717.1, D88287.1, EU498681.1, EU498714.1, X55115.1, EU498719.1, EU360958.1, M24004.1, KT240134.1, D88286.1, AB000066.1, JX475245.1, EU018143.1, EU498690.1, EU498698.1, EU145593.1, EU498682.1, HQ184200.1, HQ184203.1, AB054226.1, EU498680.1, EU360958.1, MH329286.1, AB054227.1, KP280068.1, DQ474238.1, MZ322607.1, DQ474237.1, DQ099431.1, AF015223.1, and EU498695.1; CPV: M24003, GU212792.1, M38245, MK344449, MK517973.1, and MK806279.1). The tree reconstruction was performed with MEGA 7.0 software using the neighbor-joining (NJ) method. A total of 1,000 replicates were used to generate bootstrap values.

### Virus isolation

The virus sample supernatant of FPV-DL04 was sterilized by filtration with a 0.22-μm microporous filter, synchronously inoculated into feline kidney cells (F81 cells, obtained from ATCC) in the cell division phase, gently mixed, and placed in a cell culture at 37°C and 5% CO_2_ concentration. After 1 h, the virus supernatant was discarded and replaced with the complete cell culture medium. After culturing for 72 h, the cells were placed in a −80°C refrigerator for repeated freezing and thawing twice, and the freeze-thawed culture medium was inoculated into new F81 cells. The cytopathic changes were observed after continuous blind passage for 3–4 generations, and the viral DNA was extracted for PCR detection.

### Western blotting assay

To confirm the proliferation of the FPV-DL04 strain in F81 cells, western blotting (WB) was conducted with rabbit anti-VP2 polyclonal antibodies (diluted 1:200, prepared in our laboratory) and/or with anti-β-actin mouse monoclonal antibodies (diluted 1:2,000, CWBIO, China). Protein samples were separated on 12% gels and then transferred to nitrocellulose membranes (Hybond-C; Amersham Life Sciences, UK) using a semi-dry transfer apparatus (Bio-Rad Laboratories, USA). The membranes were blocked with 5% (w/v) non-fat milk in TBST buffer (150 mM NaCl, 20 mM Tris, and 0.1% Tween-20; pH 7.6) for 2 h at 37°C and then stained 2 h at 37°C with rabbit anti-VP2 polyclonal antibodies or with anti-β-actin mouse monoclonal antibodies. After washing the membrane three times with TBST for 10 min each, goat anti-rabbit IgG secondary antibodies (1:10,000) were added, and the membrane was incubated for 1 h at room temperature. The membrane was then cleaned three times with TBST for 10 min each at room temperature. The bands were detected with the chemiluminescence kit (Thermo Fisher Scientific, USA) by using the ECL luminescence solution for chemiluminescence, exposure, and development.

### Transmission electron microscopy

To confirm the morphology and size of the FPV-DL04 strain, the harvested cell cultures were partially purified by ultracentrifugation through a 15% (wt/vol) sucrose cushion at 45,000 *g* for 3 h at 4°C. The purified sample was stained with 3% phosphotungstic acid (PTA) and observed by a transmission electron microscope (H-7500, Hitachi, Japan).

## Results

### Analysis of genetic evolution of parvovirus in clinical samples

**Figure 1:**
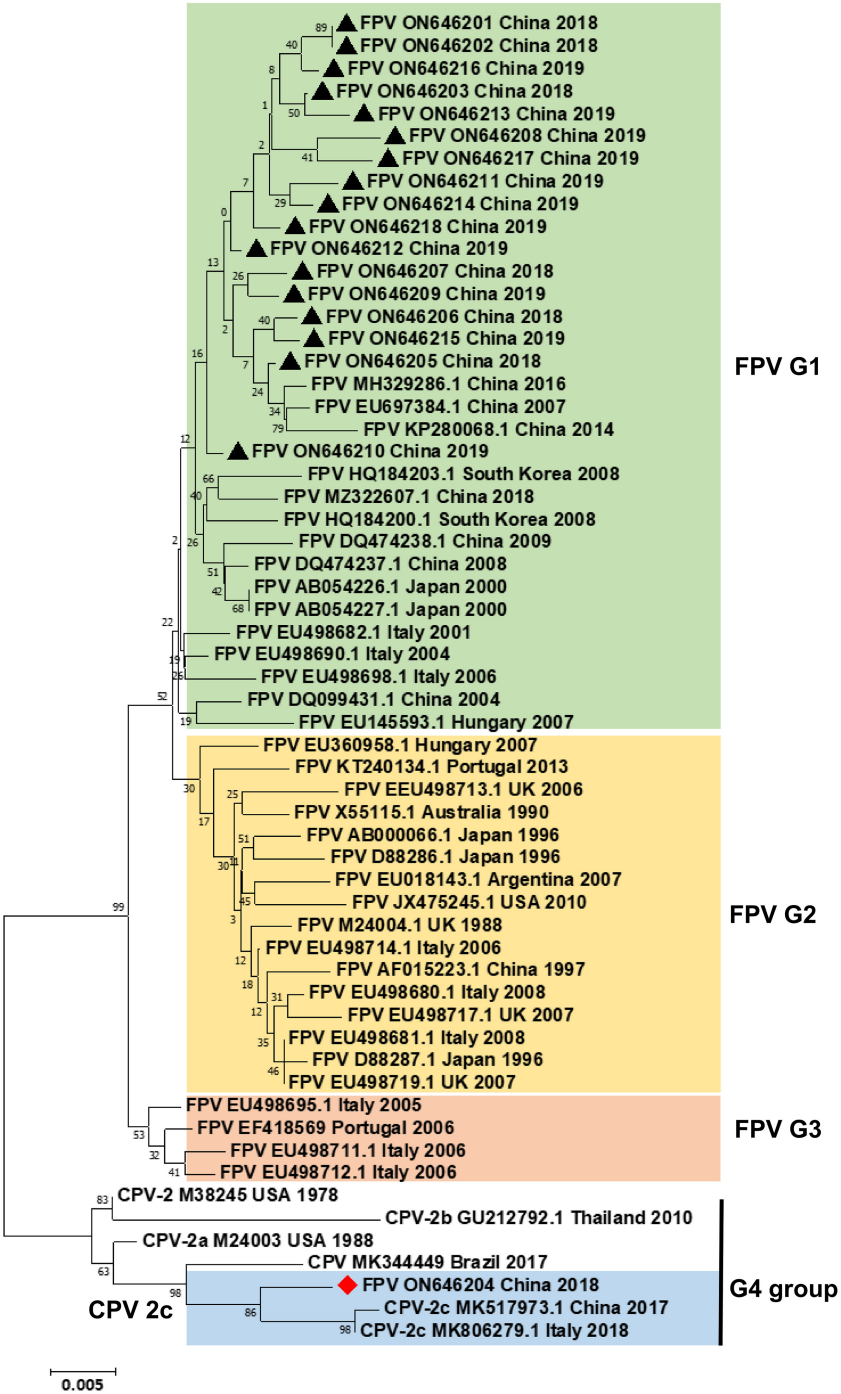
Phylogenetic tree analysis. A phylogenetic tree based on 18 VP2 gene sequences from domestic cats and 41 reference FPV/CPV strains was constructed by the maximum likelihood method using the MEGA software, version 5.1 (http://www.megasoftware.net/). ▴ indicates the FPV strains in this study. ♦ indicates the FPV DL04 strain. Horizontal branch lengths are proportional to genetic distances. Scale bars indicate nucleotide substitutions per site. Bootstrap values were calculated based on 1,000 replicates.

Fifty fecal swab samples from domestic cats with feline diarrheal disease were tested by PCR using pv-F and pv-R primers, of which 18 samples tested positive for parvovirus. Subsequently, the complete *VP2* gene sequence was amplified from 18 positive samples, cloned into the pMD18-T vector, and then sequenced. The generated nucleotide sequence was used as queries in the blast N searches and then aligned with the best hits. The phylogenetic analyses based on the complete VP2 nucleotide sequences were conducted by the NJ method in MEGA 5.1 (bootstrap replicates = 1,000). As shown in Figure 1, the phylogenetic tree contained six CPV reference sequences, 35 FPV reference sequences, and 18 cat-derived parvovirus sequences constructed from the complete *VP2* gene sequence in this study. The phylogenetic tree was divided into four groups: G1, G2, G3, and G4. Among them, both G1 and G2 groups included the FPV strains. This group mainly comprised strains from Asia and Europe. The 17 strains isolated from Dalian were in the G1 group, thus, showing that these 17 samples have obvious local origin features. The G4 group contained 3 CPV-2c strains, 1 CPV-2b strain, 1 CPV-2a strain, 1 CPV-2 strain, and 1 CPV strain, indicating that the FPV DL04 strain (GenBank number: ON646204) was located in the G4 cluster, which belongs to the same clade as the CPV-2c type virus. These results indicate that the FPV DL04 strain isolated from the cat-derived samples belonged to the CPV-2c strain.

### Analysis of amino acid mutation sites of VP2 in the FPV DL04 strain

**Figure 2:**
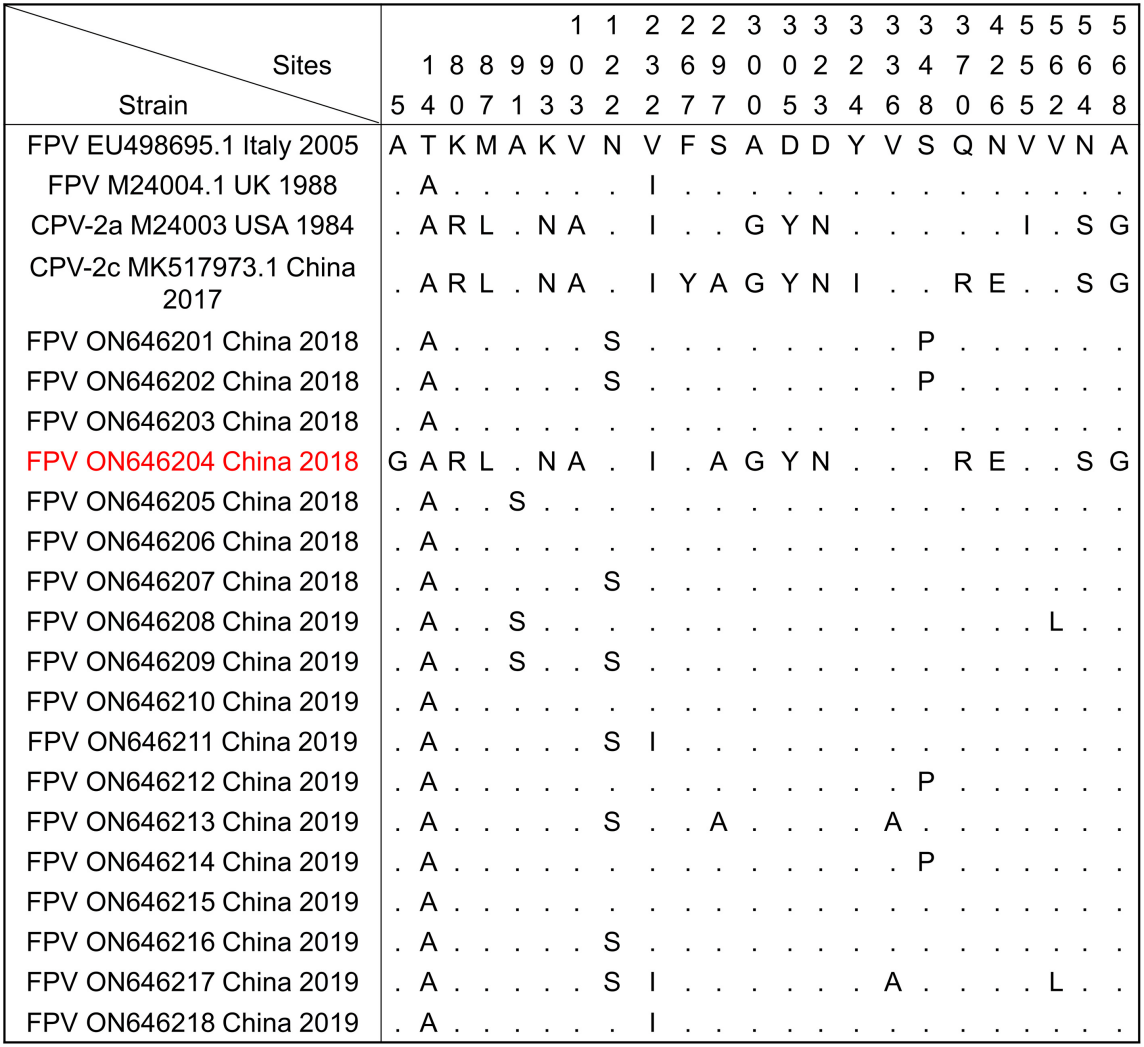
Amino acid mutations in the VP2 protein of FPV and CPVs. The amino acid mutation sites of the VP2 protein of 18 cat-derived parvoviruses obtained in this study were analyzed by MEGA 5.1 software. FPV (EU498695.1 and M24004.1), CPV-2a (M24004), and CPV-2c (MK517973.1) were used as reference sequences.

The amino acid sequences of VP2 were compared and analyzed by MEGA 5.1 software. As shown in Figure 2, the amino acid sites of 80/93/103/323/568 of the 17 sample sequences were completely consistent with the characteristic amino acid sites of FPV. The mutation sites were mainly concentrated in seven regions (A91S, N122S, V232I, S297A, V336A, S348P, and V562L). The A91S mutation was found in FPV DL05 (ON646205), FPV DL08 (ON646208), and FPV DL09 (ON646209). The N122S mutation site appeared in FPV DL07 (ON646207), FPV DL09 (ON646209), FPV DL11 (ON646211), FPV DL13 (ON646213), FPV DL16 (ON646216), and FPV DL16 (ON646216). The V122I mutation site appeared in FPV DL11 (ON646211), FPV DL17 (ON646217), and FPV DL18 (ON646218). The S297A mutation site appeared in FPV DL13 (ON646213), and it is one of the key sites for CPV-2 to acquire the ability to infect cats. The V336A mutation site appeared in DL13 (ON646213) and FPV DL17 (ON646217). The S348P mutation site appeared in FPV DL01 (ON646201), FPV DL02 (ON646202), FPV DL12 (ON646212), and FPV DL14 (ON646214). The strains FPV DL08 (ON646208) and FPV DL17 (ON646217) showed the V562L mutation site. The biological significance of these mutation sites needs further study.

**Figure 3:**
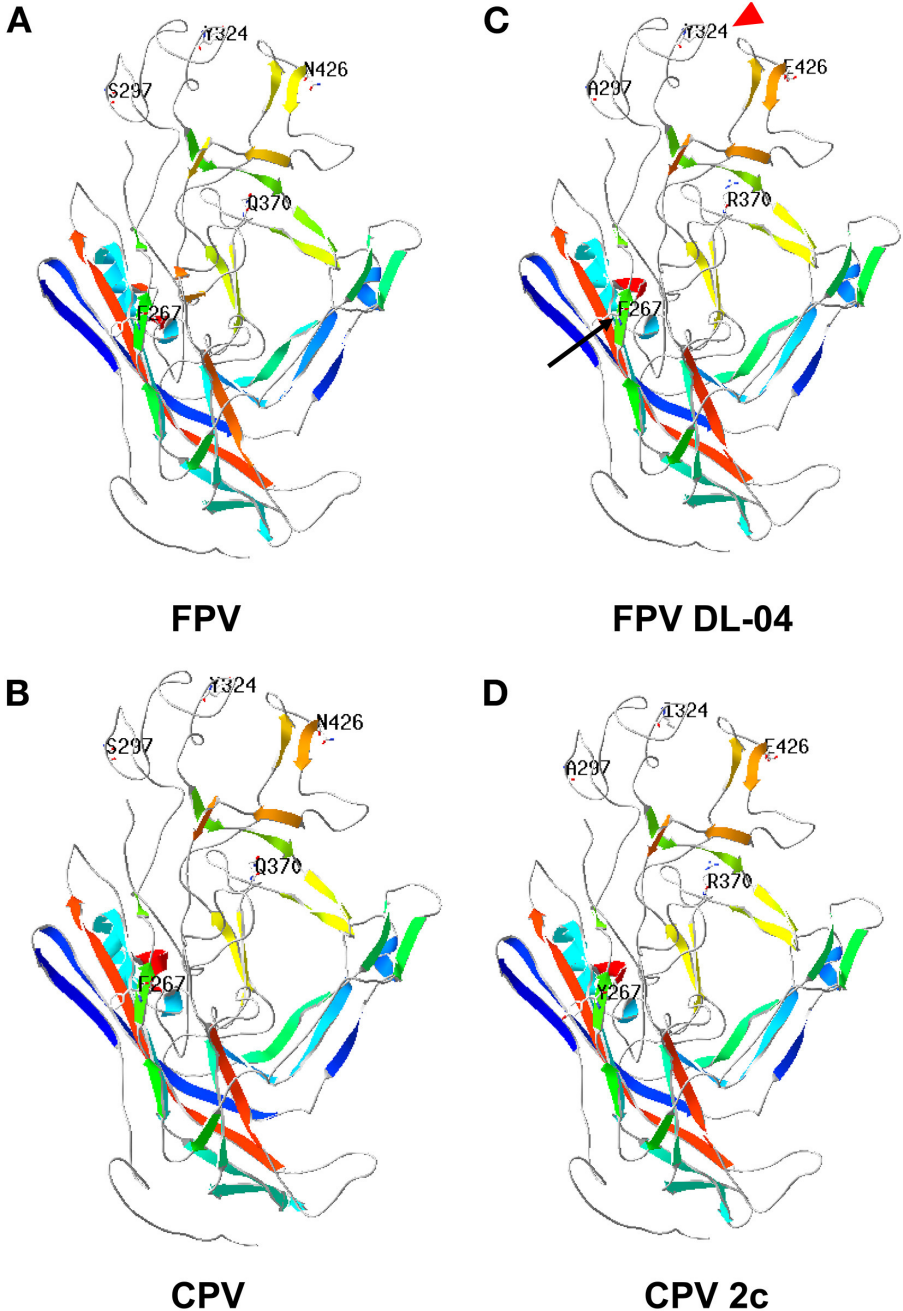
Prediction of VP2 protein structure of the FPV DL04 strain. The protein structure of the VP2 protein of the FPV DL04 strain was predicted online using the Swiss Model software (https://swissmodel.expasy.org). The CPV VP2 structure (PDB ID: 4dpv.1) was used as the template. The predicted VP2 protein structure was analyzed using the Swiss-PDB Viewer software, and the mutation sites were displayed. **(A)** FPV, **(B)** CPV, **(C)** FPV DL-04, **(D)** CPV 2c.

It is worth noting that the VP2 protein sequence of FPV DL04 (ON646204) is very close to the sequence of the CPV-2c CN/HN1708 China 2017 strain (GenBank No. MK517973.1) isolated from China and belonging to the CPV-2c type; both these sequences have four mutation points: S297A, A300G, Q370R, and N426E. In addition, A5G-specific mutation sites appeared in FPV DL04. However, this strain does not mutate at 267 and 324 sites as compared to the CPV-2c CN/HN1708 China 2017 strain. Therefore, the FPV DL04 strain is a novel CPV-2c strain from cats, and the effects of A5G, F267Y, and Y324I mutation sites on the virulence of the virus need to be further analyzed. Figure 3 shows the results of structure prediction analysis. As shown in the figure, the Y324I mutation site is located outside the capsid. The 324 site of DL04 strain was different from CPV-2c. Previous studies have revealed that this site is related to the mechanism of cross-species transmission of the virus (4). F267Y is located in the middle of the viral capsid, and its function is unknown.

### Isolation and identification of the FPV-DL04 strain

**Figure 4:**
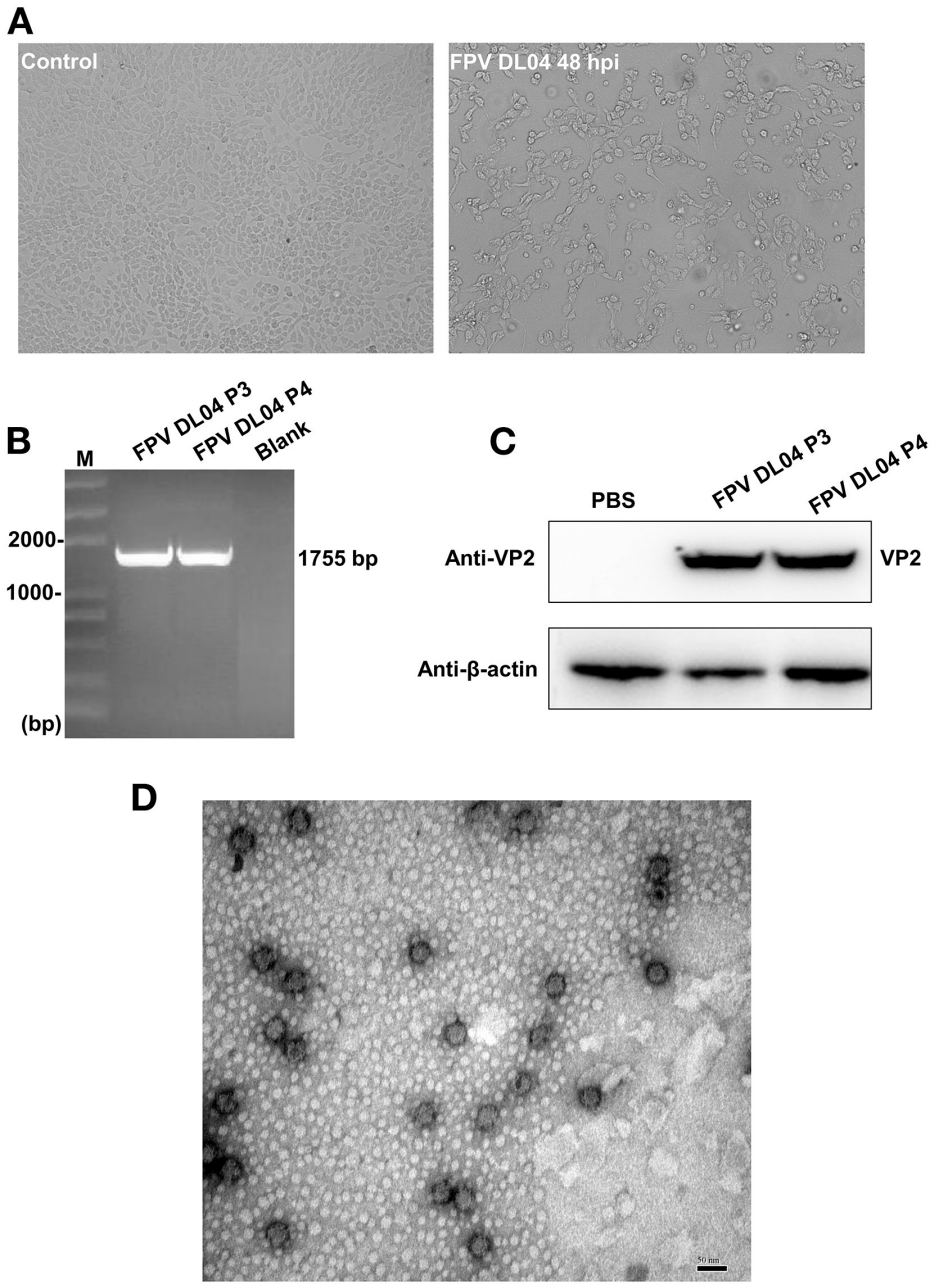
Isolation and identification of the FPV DL04 strain. **(A)** Cytopathic effect (CPE) of F81 cells infected with the FPV DL04 strain at 48 hpi ( × 100). The control F81 cells were mock infected with the isolated virus ( × 100). **(B)** VP2 PCR analysis of the total cell DNA from the third and four passages of virus-infected cells or uninfected cells. **(C)** Western blot analysis of the total cell lysates from virus-infected cells using rabbit anti-VP2 polyclonal antibodies and anti-β-actin mouse monoclonal antibodies. **(D)** Transmission electron microscopy image of the purified isolate negatively stained with 3% phosphotungstic acid. The scale bar represents 50 nm.

The FPV DL04 strain from clinical samples was blindly passed in F81 cells for three generations, and it was found that at 48 h after infection, the cells had typical parvovirus lesions such as cell rounding, decreased intercellular contact, and wire drawing, while the control group had normal cell morphology (Figure 4A), which similar to previous reports (15). Subsequently, the DNA extracted from the third and fourth generation cells infected with FPV DL04 was used as a template, and the VP2-F and VP2-R primers were used for PCR identification. The results showed that the *VP2* gene could be detected (Figure 4B). The cell lysate of the third and fourth passage cells infected with FPV DL04 for 48 h and subjected to WB analysis using VP2 polyclonal antibodies. The results are shown in Figure 4C. The VP2 band was clearly detected in both the third and fourth passage cells infected with FPV DL04. These results indicate that the FPV DL04 strain could stably proliferate in F81 cells.

To confirm the morphology and size of the FPV-DL 04 strain, the harvested cell cultures were partially purified by ultracentrifugation through a 15% (wt/vol) sucrose cushion at 45,000 *g* for 3 h at 4°C. After staining with 3% PTA, the virus morphology was observed under a transmission electron microscope. A large number of single, non-encapsulated, round virus particles were observed with diameters of ~25–50 nm; these findings are consistent with the morphological characteristics of parvovirus (Figure 4D). On the basis of the above results, we successfully isolated a cat-derived CPV-2c strain (FPV DL04 strain).

## Discussion

In the present study, 50 rectal swabs from domestic cats with diarrheal diseases were collected from a pet hospital in Dalian between 2017 and 2021 and were screened by PCR for the presence of parvovirus. Of these, 18 PCR-positive samples were subjected to *VP2* gene amplification. After obtaining the gene sequence, the homology analysis and alignment of nucleotide and amino acid sequences and phylogenetic tree construction were performed. The VP2 protein of the FPV strain contains the following key sites: 80K, 93K, 103V, 323D, and 568A. The characteristic sites of the CPV-2a VP2 protein are 426N and 297A, those of the CPV-2b VP2 protein are 426D and 297A, and those of the CPV-2c VP2 protein are 426E and 297A (1, 16, 17). The amino acid homology analysis of the VP2 protein in this study showed that the 80/93/103/323/568 amino acid positions of the 17 samples were completely consistent with the characteristic amino acids of FPV; this finding indicates that the virus in the 17 clinical samples belonged to FPV. The FPV DL04 sample showed high homology with the CPV-2c strain isolated from China, and the VP2 protein sequences of both the DL4 sample and the CPV-2c strain (GenBank No. MK517973.1) isolated from China contain S297A, A300G, and N324E as three mutation points. Thus, on the basis of these findings, the FPV DL04 strain belongs to the CPV-2c type.

Studies have shown that the original CPV-2 acquired the ability to infect cats after S297A mutation and caused diseases indistinguishable from those caused by FPV (18). CPV-2c was first reported in China in 2013 (19), and CPV-2c strains with new mutation sites were successfully isolated 4 years later, thus, indicating that CPV-2c strains are undergoing continuous genetic adaptation (15, 20–22). The VP2 protein of the CPV-2c strain isolated in this study has A5G, K80R, M87L, K93N, V103A, V232I, S297A, A300G, D305Y, D323N, Q370R, N426E, N564S, and A568G mutation sites. Among them, the A5G mutation site appeared for the first time in feline parvovirus. The A5G mutation site was first discovered in canine parvovirus VP2 in 2015, but its function is unclear (23). Moreover, the F267Y and Y324I mutation sites did not appear in the FPV DL04 strain, which was different from the CPV-2c strain isolated from China in 2017 (24). Studies have shown that the Y324I mutation affects the binding of the virus to the transferrin receptor, resulting in changes in the host range; this might be the main cause of CPV infection in cats (25). However, the FPV DL04 strain isolated from the cats in this study did not possess this mutation site, indicating that CPV-2c can also change its species characteristics through mutation at other sites, and the specific mechanism needs to be further studied.

## Conclusion

In this study, a CPV-2c strain (FPV DL04) was successfully isolated from cats. The amino acid sequence alignment analysis showed that the S297A and A300G mutation sites unique to CPV-2c appeared in the FPV DL04 strain, but the F267Y and Y324I mutation sites did not appear. The strain also showed the A5G mutation site. This study provided new important findings regarding the evolution of parvovirus infection in domestic cats.

## Data availability statement

The datasets presented in this study can be found in online repositories. The names of the repository/repositories and accession number(s) can be found in the article/supplementary material.

## Author contributions

ZJ and YanmW conceived the project, analyzed the data, and revised the manuscript. ZJ and YanqW designed and performed the experiments, analyzed the data, and wrote the manuscript. ZJ, PJ, YanmW, and FH wrote sections of the manuscript. All authors contributed to the article and approved the submitted version.

## Funding

This study received funding from Gansu Provincial Youth Fund (Grant Number 21JR7RA482) and the Open Project of the State Key Laboratory of Animal Genetic Engineering Vaccines (Grant Number AGVSKL-ZY-201806).

## Conflict of interest

The authors declare that the research was conducted in the absence of any commercial or financial relationships that could be construed as a potential conflict of interest.

## Publisher's note

All claims expressed in this article are solely those of the authors and do not necessarily represent those of their affiliated organizations, or those of the publisher, the editors and the reviewers. Any product that may be evaluated in this article, or claim that may be made by its manufacturer, is not guaranteed or endorsed by the publisher.
